# Current Evidence-Based Clinical Nutritional Approaches in Lipedema: A Scoping Review

**DOI:** 10.1093/nutrit/nuaf203

**Published:** 2025-11-25

**Authors:** Büşra Atabilen Pınar, Menşure Nur Çelik, Hilal Betül Altıntaş Başar, Duygu Ağagündüz, Oya Berkay Karaca

**Affiliations:** Department of Nutrition and Dietetics, Faculty of Health Sciences, Karamanoğlu Mehmetbey University, Karaman, 70100, Türkiye; Department of Nutrition and Dietetics, Faculty of Health Sciences, Ondokuz Mayıs University, Samsun, 55200, Türkiye; Department of Nutrition and Dietetics, Faculty of Health Sciences, Erzincan Binali Yıldırım University, Erzincan, 24100, Türkiye; Department of Nutrition and Dietetics, Faculty of Health Sciences, Gazi University, Ankara, 06490, Türkiye; Department of Gastronomy and Culinary Arts, Faculty of Fine Arts, Çukurova University, Adana, 01330, Türkiye

**Keywords:** lipedema, obesity, clinical nutrition therapy, nutritional supplements

## Abstract

Lipedema, a chronic condition primarily affecting women, is characterized by abnormal subcutaneous fat accumulation and swelling in the extremities (while sparing the hands, feet, and trunk). This disease is associated with genetic predisposition, hormonal imbalances, impaired lymphatic function, and vascular dysfunction. Lipedema does not directly cause weight gain, but excess weight can worsen symptoms and accelerate disease progression. Bariatric surgery is considered a treatment option for body weight management and reduction of subcutaneous fat; however, reported studies have indicated that this treatment cannot reduce localized fat accumulation or fat cell hypertrophy or alleviate pain symptoms. Although no proven dietary treatment currently exists, nutrition plays a key role in managing lipedema. Certain dietary approaches such as ketogenic, low-carbohydrate, and modified Mediterranean diets have been explored for weight management and inflammation reduction in lipedema, with studies showing positive effects on body composition and pain. However, according to the current literature no evidence-based nutritional treatments or nutritional supplements are effective in this patient group. Nutritional therapy in lipedema is complicated by frequent comorbidities; therefore, precision nutritional therapy should be planned by evaluating the causes and consequences of the disease. In this review, we evaluated reported studies of current evidence-based clinical nutritional approaches to lipedema treatment.

## INTRODUCTION

Lipedema is a chronic condition that occurs predominantly in women. Lipedema is characterized by abnormal subcutaneous fat accumulation and swelling in the extremities, which disrupts fat metabolism.^1^ Lipedema affects only the extremities, from the hips to the lower legs in the legs and from the shoulder area to the forearms in the arms. The hands, feet, trunk, neck, and head remain unaffected.^2^ Additional clinical features of lipedema include quick bruising after minor trauma, sensitivity, and sensations of heaviness, pressure, and spontaneous pain.^2^^,^^3^ The effects of lipedema include accompanying pain and physical immobility, and this disease can negatively affect patient mental health by causing the development of depression.^4^ Due to the lack of well-recognized diagnostic criteria and the difficulties in distinguishing lipedema from obesity or lymphedema, lipedema is sometimes misdiagnosed, with symptoms misinterpreted as lymphedema, and the incidence of lipedema is still unknown.^5^ The overall prevalence of lipedema in the general population has been reported to be as low as 0.1%, although the prevalence in outpatient clinics is estimated to be between 7% and 10%. In contrast, prevalence rates in hospitalized patients range from 8% to 18%.^6^

This scoping review was conducted to identify current evidence-based clinical nutritional approaches in lipedema and to reveal gaps in the existing literature. A literature search was performed using the Web of Science, Google Scholar, and ScienceDirect databases. No strict publication date limits were applied; however, priority was given to English-language studies published in recent years. The search strategy used keywords such as “lipedema,” “nutrition,” “dietary intervention,” “clinical nutrition,” and “evidence-based,” combined using appropriate Boolean operators (AND, OR). Inclusion criteria consisted of peer-reviewed research and review articles focusing on nutritional approaches in lipedema with full-text availability. In addition, a limited number of book chapters and reliable internet sources were also consulted to complement the scientific literature.

Proposed mechanisms underlying the excess fat in lipedema include genetic factors, hormonal imbalances, lymphatic system impairments, and vascular dysfunction. Genetically, elevated expression of genes linked to cell proliferation and mitotic clonal expansion may cause hyperplasia.^7^ One study revealed that adipocytes in lipedema patients had much higher expression of the leptin gene, peroxisome proliferator-activated receptor gamma (*PPAR-γ*), and that adipogenic differentiation was more likely in adipose-derived stem cells.^8^ A mutation in the aldo-keto reductase family one member C1 (*AKR1C1*) gene or in the pituitary-specific positive transcription factor 1 (*PIT1*) gene for expression of growth and sex hormones can also cause lipedema.^9^ Furthermore, several genes have been found to be differently expressed and implicated in the processes that cause lipedema. Adipogenesis involves several genes that control the generation of mature adipocytes from mesenchymal stem cells, including protein kinase cyclic guanosine monophosphate (cGMP)–dependent 2 (*PRKG2*), mesenteric estrogen-dependent adipogenesis (*MEDAG*), colony-stimulating factor 1 receptor (*CSF1R*), bicaudal C family RNA binding protein 1 (*BICC1*), erythroblastic leukemia viral oncogene (erb)-b2 receptor tyrosine kinase 4 (*ERBB4*), and acid phosphatase 5 (*ACP5*).^10^ In addition to recognizing genetic involvement, identifying specific microRNAs (miRNAs) in lipedema-affected adipose tissue has provided insights into the molecular mechanisms of this disease. The miRNA hsa-let-7g-5p has been shown to be upregulated and 12 other miRNAs to be downregulated in lipedema tissue. These differentially expressed miRNAs are involved in processes such as cell proliferation, adipogenesis, and extracellular matrix remodeling.^11^

From a hormonal perspective, unbalanced estrogen-mediated processes linked to region-specific sympathetic innervation of subcutaneous adipose tissue and energy balance may have an impacts on the pathogenesis of lipedema.^5^^,^^12^ The hormonal background of this disease is demonstrated by the fact that it typically manifests during puberty, pregnancy, and menopause.^13^ Lipedema, characterized by an unequal buildup of body fat in the lower body, is thought to be associated with dysregulation of estrogen signaling. In particular, changes in the balance of the ERα and ERβ estrogen receptors may affect lipid storage, lipolysis, and metabolic processes, potentially triggering the development of lipedema during periods of hormonal changes.^14^ An increased number of CD163-positive M2 macrophages has been observed in the adipose tissue of lipedema patients, potentially because of chronic estrogen exposure. These M2-polarized macrophages, characterized by expression of the scavenger receptor CD163, have been shown to support adipocyte lipid accumulation and hypertrophy.^15^ Although M2 macrophages are typically associated with anti-inflammatory functions and tissue repair, their excessive activation can lead to sustained production of transforming growth factor-beta (TGF-β) and various growth factors, promoting fibrosis.^16^ Major microvascular malfunction in the blood and lymphatic vessels is the subject of another pathophysiological theory. Vascular dysfunction and increased inflammation can occur due to remodeling of the extracellular matrix by the excessive growth of fat cells and the loosening of endothelial cell tight junctions.^7^ Alterations in vascular and lymphatic vessel function are central to the inflammatory pathology of lipedema. These changes disrupt tissue fluid homeostasis and facilitate the accumulation of immune cells and inflammatory cytokines, thereby promoting a persistent pro-inflammatory microenvironment.^17^ At higher disease stages greater infiltration of immune cells, especially macrophages, is exhibited. A pro-inflammatory shift has been observed in immune cell populations, which correlates with increased expression of inflammatory cytokines and activation markers.^18^

An observational study on a large Italian population with lipedema has provided valuable insights into the inflammatory aspects of the condition. In this study investigators found elevated levels of C-reactive protein at more severe clinical stages, indicating increased systemic inflammation.^19^ Excessive collagen deposition has also been noted as a type of extracellular matrix remodeling. Furthermore, there was an observed decrease in the capacity of lymphatic capillaries to receive interstitial fluid, which leads to interstitial fluid leakage and lymphatic dysfunction. The extra interstitial fluid that surrounds the fat cells serves as a source of nutrition, which promotes the abnormal growth of fat cells.^7^ In addition, it has recently been suggested that increased intestinal permeability and accumulation of bacterial lipopolysaccharides in lower-body fat depots may play a role in the formation of lipedema by causing inflammation. Increases of intestinal permeability and lipopolysaccharides may play a role in comorbidities of lipedema such as asthma, allergies, Hashimoto thyroiditis, and polycystic ovary syndrome.^20^ Felmerer and colleagues reported that fibrosis and increased adipocyte hypertrophy in lipedema patients, along with macrophage presence and abnormal lipid metabolism, are potentially linked to metabolic issues and insulin resistance.^21^ In an investigation of the adipogenic gene profile of lipedema, it was discovered that 5 genes had variable expression, particularly CCAAT/enhancer-binding protein delta (*CEBPD*), a transcription factor known to be involved in inflammatory responses and estrogen regulation, and Kruppel-like factor (*4KLF4*), which is essential for the development of skin barrier function and plays a role in lipid metabolism and adipogenesis regulation.^21^

The formation of lipedema step by step includes the following mechanisms:

1. Genetic Predisposition: Lipedema is considered a polygenetic disease that mainly affects women and is thought to have a familial inheritance pattern.
2. Hormonal Effects: A key role in lipedema pathogenesis is played by estrogen. It influences lipolysis and lipogenesis, leading to regional fat accumulation.
3. Vasculopathy: Lipedema is associated with microcirculatory disorders and endothelial dysfunction. These factors increase vascular permeability, causing edema.
4. Lymphangiopathy: Impaired lymphatic function leads to fluid accumulation and fat tissue expansion, worsening lymphatic insufficiency.
5. Adipocyte Hyperproliferation: Excess fat compresses blood and lymphatic vessels, causing hypoxia and inflammation. These processes promote further adipocyte growth, creating a vicious cycle.
6. Neuropathy: Inflammation of peripheral nerves and sympathetic innervation dysfunction cause pain and sensitivity.
7. Inflammation and Fibrosis: Hypoxia and adipocyte necrosis trigger inflammatory cytokine release. This leads to fibrosis and further lymphatic drainage impairment. These steps explain the complex pathophysiology of lipedema and its self-perpetuating mechanisms.^22^

The diagnosis of lipedema is usually made after the exclusion of similar diagnoses, such as lymphedema and obesity. A clinical diagnosis of lipedema must be made. For a differential diagnosis, instrumental examination techniques (duplex sonography, ultrasound, and other imaging methods) and laboratory parameters may be employed.^2^ Additionally, non-contrast magnetic resonance lymphography is a noninvasive technique that provides valuable information for differential diagnosis and assessment of lymphatic appearance in patients with lipedema and lipolymphedema, while also enabling objective measurements of enlarged limbs to monitor treatment efficacy.^23^ A diagnosis of lipedema is confirmed when a patient has an excessive growth of adipose tissue on the limbs that is bilaterally symmetrical but absent from the hands and feet, along with tissue tenderness, a tight feeling, and an excessive tendency toward hematoma formation.^24^ Specific characteristics of lipedema, including that it usually begins during puberty, is associated with easy bruising, and presents with a sharp border at the ankle (cuff sign), are important in the differential diagnosis of lipedema.^6^

The differences between lipedema and lymphedema are that lymphedema typically begins in the toes, whereas swelling in lipedema patients usually affects the thighs first, and the Stemmer sign (inability or difficulty in pinching and lifting the thickened skin at the base of the second toe) is negative in the case of lipedema.^13^ In lipedema, damage to small blood vessels causes capillary fragility and the accumulation of protein-rich fluid between cells, leading to edema and subcutaneous bruising. This fluid buildup disrupts lymphatic drainage, potentially resulting in lipolymphedema,^25^ an additional factor that is associated with the occurrence of a compromised lymphatic system and complicates the clinical diagnosis of lipedema.^26^

The 3 stages of lipedema are distinguished by progressive alterations in the structure of the skin surface when assessed in terms of clinical evaluation. In stage I, small nodules and reversible edema are present. In stage II, nodules have increased in size (walnut size), and edema is reversible or irreversible. Stage III is characterized by malignant adipose tissue, macronodular alterations, concomitant lymphedema, and perhaps a positive Stemmer sign. Regular clinical follow-up should include standardized anthropometric measurements, including body weight, body mass index (BMI), waist-to-hip ratio (WHR), waist-to-height ratio, and limb volume and circumference.^24^

In the multimodal management of lipedema, the main goals are reducing discomfort and heaviness, contouring the afflicted limbs, controlling weight, and increasing mobility to enhance quality of life.^27^ Compression therapy is performed to reduce pain in the affected extremities when lipedema is identified. Medicinal pain therapy may be considered. Additional lymph drainage combined with additional therapeutic approaches may be used to treat the cardinal symptom of pain if compression therapy is unsuitable in a particular situation or does not produce pain alleviation.^2^ Physical exercise constitutes a fundamental component of the comprehensive and multidisciplinary management of lipedema. Exercise contributes to numerous benefits, including improved mitochondrial function, increased lymphatic drainage, and reduced inflammation. Structured and individualized physical activity, especially water-based activity, offers both physical and psychological benefits in lipedema management.^28^ Other therapeutic strategies for lipedema may include postural correction, core stabilization, progressive resistance training, gait retraining, and diaphragmatic breathing, all aimed at improving neuromuscular function, enhancing lymphatic return, and promoting parasympathetic nervous system activation.^29^ There are also alternative treatments for lipedema, such as liposuction. The results of a systematic review showed that liposuction may be an alternative treatment method in lipedema patients who do not benefit from compression therapy.^30^

Bariatric surgery (BS) has been tried as a treatment option for patients with lipedema accompanied by obesity. However, it has been reported that BS is effective in providing weight loss but has a limited effect on lipedema fat.^31^ So, lipedema or pain related to lipedema cannot be cured by BS.^32^ Almost all lipedema patients report having trouble embracing their bodies, particularly their legs. Additionally, psychological evaluations revealed that a notably greater percentage of lipedema patients reported experiencing physical or sexual abuse. For this reason, psychosocial support should also be added to the treatment plan.^33^

**Figure 1.**
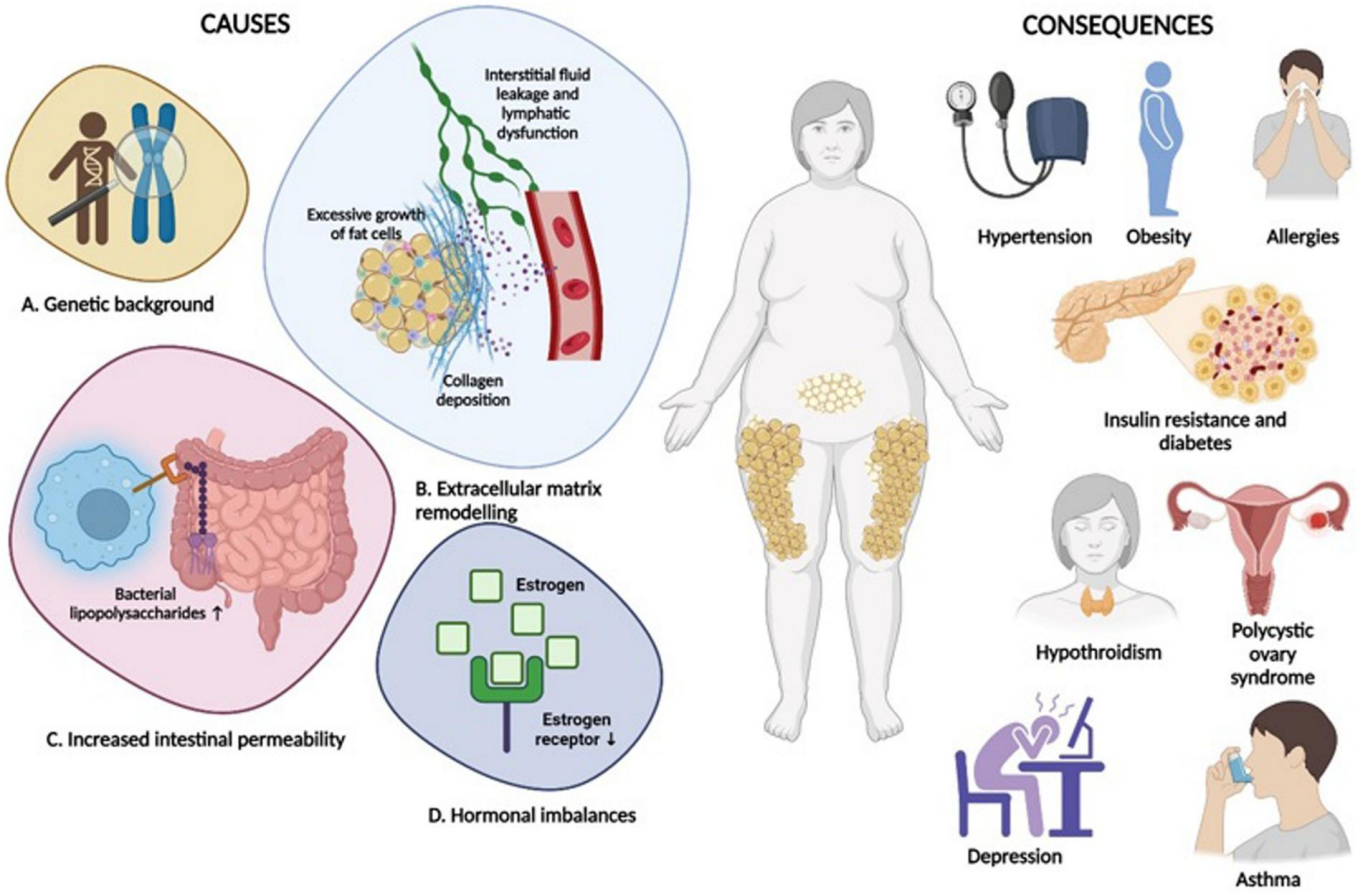
Some Causes and Consequences for Lipedema

Nutrition and dietary approaches in lipedema provide treatment options that should be considered in alleviating or improving disease symptoms. However, nutritional therapy becomes complex due to comorbidities such as allergies, hypothyroidism, depression, hypertension, etc.^20^^,^^34^ Therefore, when planning nutritional therapy in this patient group, it is important to evaluate both the factors that cause the disease and the comorbidities that occur because of the disease and to plan individualized nutritional planning. The certain causes and consequences that should be taken into consideration when creating a nutrition protocol for lipedema are summarized in Figure 1.

Although weight gain exacerbates the symptoms of lipedema, there is no proof that lipedema causes weight gain. Therefore, weight management as a treatment option may lead to significant improvements in symptoms and prevent disease progression.^33^ Dietary models such as the ketogenic diet,^35–37^ modified Mediterranean ketogenic diet,^38–41^ low-carbohydrate diet,^42–44^ and modified Mediterranean diet^45^ have been investigated as options for weight management and reducing inflammation in lipedema. Additionally, dietary supplements may be useful in disease management due to anti-inflammatory and antioxidant effects that lead to reduced edema and pain. In particular, omega-3 fatty acids and vitamin C are recommended in the treatment of lipedema.^46^ The effects of dietary supplements such as carnitine, conjugated linoleic acid, and chromium, which may be important in the treatment of lipedema by accelerating fat breakdown, are also being evaluated.^47^ However, owing to the frequency of resistance to lifestyle changes involving dietary adjustments and increased exercise, nutritional approaches to managing lipedema are not always agreed upon by medical professionals.^29^ There are even sources stating that dietary adjustments will not reduce the fat tissue accumulated in the lower extremities^48^^,^^49^ but can only alleviate symptoms such as pain, edema, and inflammation.^49^

## NUTRITIONAL STRATEGIES FOR LIPEDEMA

A crucial part of the treatment of any disease is medical nutrition therapy. According to current literature, there is no proven nutritional treatment for lipedema. Due to the fibrotic component in the loose connective tissue in lipedema, weight loss through diet is more difficult in this patient group.^29^ However, studies related to nutritional strategies to treat symptoms and slow down progression in lipedema patients are continuing.^38^^,^^39^^,^^43^^,^^44^

**Figure 2.**
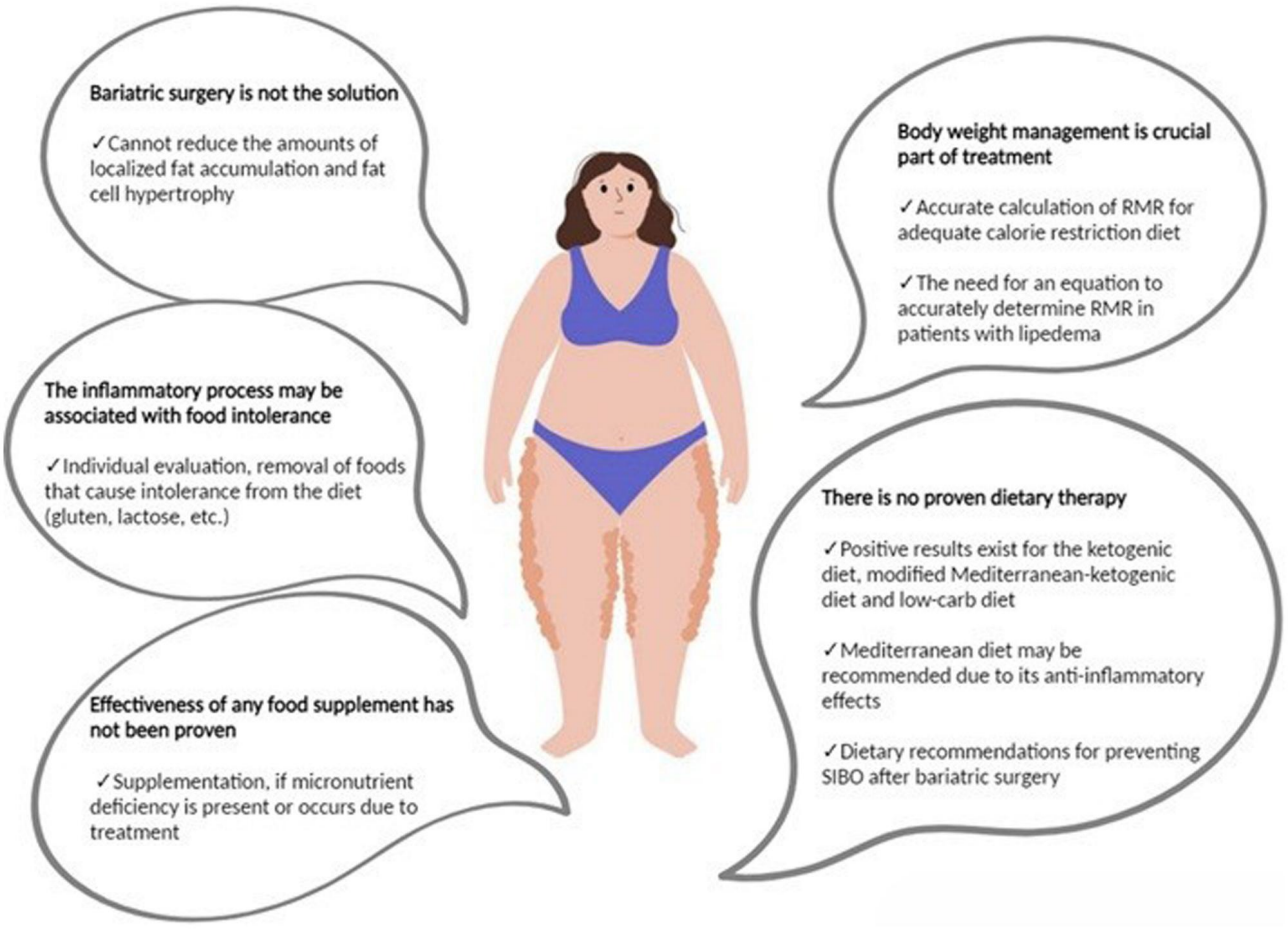
Nutritional Strategies in Patients with Lipedema

Nutritional strategies based on current evidence in lipedema, discussed above, such as weight management in lipedema, especially in the context of obesity; nutritional therapy in patients undergoing BS; effects of different dietary models in the treatment of lipedema; the role of food intolerance; and dietary supplements are summarized in Figure 2.

### Body Weight Management

Approved clinical criteria are used to diagnose lipedema.^24^ Among clinicians, one of the fundamental criteria—the existence of a visual disparity in body fat distribution—can be arbitrary and unreliable. Therefore, among disorders marked by excess fat in the lower limbs, lipedema is particularly hard to identify.^27^

Dual-energy X-ray absorptiometry (DXA) is a low-radiation, noninvasive imaging technique that is frequently used to determine the composition of soft tissues and the mass of bones throughout the body.^50^ In recent years, DXA has emerged as the standard clinical method for determining the distribution of body composition (BC) and fat mass (FM).^51^ The use of DXA is proposed as a diagnostic tool for lipedema because of its ability to make body fat distribution disproportionality quantifiable and objective.^1^ Recognizing this condition in individuals with excessive limb fat accumulation is very crucial to prevent needless therapies, manage patient expectations through education, and deliver the right therapy.^52^ It has been proposed that DXA may enable adequate early therapy and avoidance of late consequences of the disease in people with suspected lipedema.^1^

The majority of people with lipedema are overweight or obese, and losing weight is a crucial part of their treatment.^39^ Weight-loss diets help these patients move more freely and lower their chance of getting secondary lymphedema.^53^ Calorie restriction used to treat obesity in lipedema patients is crucial in clinical practice to accurately measure the energy needs of individuals with lipedema.^54^ However, a case report has demonstrated that lipedema can persist in women who believe that lipedema is due to obesity despite severe protein and caloric malnutrition during their weight-loss efforts, and that BMI may be a misleading guide for diagnosing lipedema, malnutrition, and obesity.^55^ To maintain weight and body fat loss in patients with lipedema, adequate dietary energy intake is crucial. In these patients an adequate calorie-restricted diet can be implemented with the help of an accurate calculation of the basal metabolic rate (BMR).^43^

The BMR, or the amount of energy the body uses at rest to sustain important functions for a full day, is one of the components of total energy expenditure, accounting for 60%-75% of its value during an inactive period.^56^ The BMR can be determined with predictive equations, bioelectrical impedance analysis, and indirect calorimetry (IC),^57^ which is considered the gold standard for determining the real resting metabolic rate, particularly in individuals with acute or chronic illnesses.^58^ However, using IC in a therapeutic setting is costly, necessitates specialist equipment, and is not generally accessible.^59^

Approximated equations are the most commonly used technique for estimating BMR when BMR cannot be obtained via IC.^60^ However, in the case of chronic conditions, the predictive equations may not be correct, even though they are reasonably dependable in healthy persons.^61^ Based on anthropometric measurements like height, weight, or lean body mass, estimated equations offer a rather rapid and simple way to determine BMR.^62^

Age, height, sex, body weight, physical activity, and body composition—including lean body mass and body fat—all have a significant impact on BMR,^63^ as do other variables like diet, obesity, hormonal status, ethnicity, and genetic and environmental factors.^64^ The quantity of body fat and lean body mass are reported to have a substantial impact on BMR.^65^ In patients with lipedema, the disproportionate amount of body fat in the legs relative to the upper body suggests that total body fat may not be a particularly effective metric for determining BMR.^66^

Estimation of the BMR is done using the following equations: Harris–Benedict, Mifflin, Bernstein, Owen, Food and Agriculture Organization/World Health Organization (FAO/WHO), Cunningham, Müller, Korth, Lazzer, Huang, Henry, and Institute of Medicine (IOM).^67^ According to a study, the BMR that was computed using equations in patients with lipedema was 60% less consistent than the actual BMR.^66^ According to the statement, this circumstance suggested the need for a new equation for individuals with lipedema.^66^

The study by Karagun and Baklaci found that BMR calculated using the Mifflin–St Jeor and Harris–Benedict equations showed strong agreement, but agreement was poorer in obese patients.^68^ However, these formulas may not be reliable for lipedema patients due to uneven fat distribution, as they do not account for body composition or fat distribution factors.^7^

In clinical practice, waist circumference can be used to track the effectiveness of obesity treatment. It is advised to measure the WHR to identify the type of obesity and the distribution of body fat percentages.^69^ However, because of the disproportionate amount of body fat in the thighs and hips relative to the waist, it has been suggested that the WHR may not be helpful in clinical practice for treating patients with lipedema.^70^

When access to the gold standard IC is restricted, a new formulation was required to calculate BMR because the existing equations do not have high enough compatibility rates for calculating BMR in patients with lipedema. Consideration was given to reducing the consequences of the disproportion between the upper and lower regions, which is a common characteristic of people with lipedema. Lean body mass, body fat mass, total body water, visceral fat level, waist, hip, WHR, height, weight, body mass index (BMI), and age parameters are all included in the newly created formula. The developed formula is given below.^71^


BMR=(0.0472×PC1st+0.0452×PC2st+0.0509×PC3st-0.600)×310.5558+1693.5234if0.0482×PC1st+0.0452×PC2st+0.0509×PC3st-0.600≤-0.0567BMR=(0.2160×PC1st+0.2184×PC2st+0.2116×PC3st+0.4945)×310.5558+1693.5234 otherwisePC1st=[0.2328×age-0.2903×height+0.2936×weight+0.3674×BMI+0.2726×LBM+0.3374×PBF+0.3294×MBF+0.2066×TBW+0.3174×VFL+0.2962×waist+0.2795×hips+0.1853×WHR-109.5763]/34.5448PC2st=[0.4479×age+0.4934×height-0.2250×weight-0.0695×BMI-0.2927×LBM+0.0497×PBF-0.1349×MBF-0.3635×TBW+0.0776×VFL+0.0535×waist-0.3113×hips+0.3951×WHR-109.9039]/34.5661PC3st=[0.3150×age-0.1447×height-0.0650×weight-0.0215×BMI-0.1423×LBM+0.1300×PBF-0.0030×MBF-0.2148×TBW+0.3330×VFL-0.3125×waist+0.2579×hips-0.7189×WHR-108.9802]/34.5062,

where BMI is body mass index; BMR, basal metabolic rate; LBM, lean body mass; PBF, percentage body fat; MBF, mass of body fat; PC, principal component; TBW, total body water; VFL, visceral fat level; WHR, waist–hip ratio.

### Bariatric Surgery and Medical Nutritional Therapy

Several diseases are linked to obesity, making it a factor that has a detrimental impact on health. Bariatric surgery (BS) is used to lessen the impact of obesity and associated disorders in patients who are unable to lose weight without surgery.^72^ Despite the variety of BS options, patients may encounter some difficulties.^73^

Although BS has been demonstrated not to affect the symptoms of lipidemia sufferers, lipedema can be identified during BS because the great majority of people with this condition are obese. Before undergoing surgery, it is crucial to carefully review the symptoms and assess the diagnostic standards this time. An essential part of postoperative care in BS candidates is evaluating nutritional status before surgery.^74^ Compared to people with normal body weight, patients with severe obesity are known to frequently display micronutrient deficiencies (MD).^75^ Preventing MD after BS is thought to depend on the nutritional state of BS patients being evaluated and corrected prior to the treatment.^76^

While there are several forms of BS, from a nutritional standpoint, its impacts on nutritional status are mostly associated with decreased stomach capacity and nutrient absorption.^77^ In the majority of BS, the stomach is reduced in size, and/or a tiny gastric pouch is created. In the initial days following surgery, eating solid foods is extremely difficult or impossible due to the tiny volume and postoperative stomach edema.^78^ Thus, most postoperative dietary programs advise a liquid or extremely soft diet in the first few days following surgery and a very gradual increase in food firmness in the first few weeks to avoid or reduce vomiting and regurgitation.^79^

Obesity and lipedema are closely related. Nearly 90% of patients with lipedema at specialty clinics are obese (BMI ≥30 kg/m^2^).^29^ According to reports, BS cannot reduce the amounts of localized fat accumulation and fat cell hypertrophy in the inflammatory subcutaneous fat tissue of individuals with lipedema.^7^

Obesity or other causes of lower extremity enlargement might be mistaken for lipedema, a disorder characterized by abnormally symmetrical bilateral lower extremity or trunk fat tissue.^80^ When individuals undergo BS, making a precise diagnosis may be disregarded and neglected.^81^ There have been reports that after BS, people with lipedema may continue to have broad trunk and lower extremity fat that is resistant to weight loss.^82^

Even after losing weight, patients who have been diagnosed with lipedema following BS have not shown improvement in the pain sensations that are typical of the condition. Because the causes of lipedema and obesity are different, lipedema patients require a different kind of care. It is always important to assess obese patients for lipedema and discomfort. If lipedema is diagnosed concurrently, BS should be carried out only if diet and exercise have failed, the patient BMI is greater than 40 kg/m^2^ and the patient has been advised that pain may continue after BS.^32^

Nonetheless, in 1 study the leg volumes of women with lipedema were compared before and after Roux-en-Y gastric bypass (RYGB) or sleeve gastrectomy (SG). In contrast to earlier findings, it was discovered that SG and RYGB significantly reduced the volume of the thighs in lipedema patients.^83^

### Effects of Different Dietary Patterns

Notably, nutritional management is seen to be a treatment option for preventing morbidities and preserving health in lipedema patients. Therefore, the development of precision-nutrition therapeutic strategies prepared by considering the pathophysiology and morbidities of the disease and will provide adequate nutrition is very important in managing symptoms and slowing down the progression of lipedema. In this section we provide an overview of the different dietary treatments frequently recommended for lipedema.

#### Ketogenic Diet

The ketogenic diet is a nutritional model that emphasizes moderate protein, high fat, and low carbohydrate intake. Reducing daily carbohydrate intake to less than 50 g is the general aim of the ketogenic diet. The amount of protein consumed is moderately limited to less than 1 g/kg of body weight.^84^ The ketogenic diet is recognized as an effective dietary intervention for refractory epilepsy and is currently being explored as a potential treatment for various conditions, including obesity and cancer.^85^ The ketogenic diet has also been reported to be an important nutritional model in reducing weight and excess adipose tissue accumulation, reducing pain, and improving quality of life in patients with lipedema.^39^^,^^41^^,^^54^^,^^86^ Mechanisms such as satiety induced by the decrease in insulin and leptin levels, reduction in inflammation and oxidative stress because of mitochondrial respiration and inhibition of NLR family pyrin domain containing 3 (*NLRP3*), and inhibition of fibrogenesis by increasing adiponectin levels with ketogenic diet intervention may be important in the treatment of lipedema.^37^ As previously mentioned, insulin resistance is associated with increased adipocyte hypertrophy and fibrosis, which are important in the development of lipedema.^21^ Furthermore, the upregulated expression of insulin receptor (*INSR*), glucose transporter type 4 (*GLUT4*), and lipoprotein lipase (*LPL*) genes in lipedema adipocytes indicates a potential role of enhanced insulin signaling in promoting adipocyte hypertrophy associated with lipedema.^87^ Mechanistically, insulin resistance in adipocytes leads to increased production of chemokine monocyte chemoattractant protein-1 (MCP-1), which promotes monocyte recruitment and the activation of proinflammatory macrophages.^88^ However, the ketogenic diet improves hepatic insulin sensitivity and lipid metabolism by reducing carbohydrate availability, thereby lowering hepatic triglyceride synthesis and enhancing mitochondrial function via increased β-oxidation and ketogenesis. Therefore, the ketogenic diet may mechanistically help alleviate insulin resistance, which is proposed as one of the contributing factors in lipedema.^89^ In addition, carbohydrate restriction diminishes neuronal excitability, which may lead to a reduction in pain perception, inhibition of glycolysis, attenuation of inflammation, and an increase in levels of adenosine, a natural analgesic.^90^ Also, carbohydrate restriction may decrease the fluid load on the lymphatic system, thereby lowering tissue water content and preventing edema.^37^

Some studies on the effects of the ketogenic diet on lipedema are given in Table 1. In 1 study,^54^ 9 female patients with lipedema were given a eucaloric ketogenic diet for 7 weeks. Then, participants were given a Nordic diet intervention for 6 weeks. At the end of the study, there was a substantial reduction in body weight both at the end of the 7th week (*P* < .001) and the 13th week (*P* < .001). Although the decrease in pain was significant at the end of the 7th week (*P* = .018), the pain returned to baseline levels at the end of the 13th week. In the case report by Cannataro et al., a hypocaloric ketogenic diet intervention was performed for 22 months in a 32-year-old woman with lipedema. At the end of the study, weight loss of up to 41 kg and reductions in body circumference measurements and pain were recorded.^86^ In the S2k guideline led by the German Society of Phlebology and Lymphology (DGPL), it was emphasized that “a ketogenic diet (hypocaloric if necessary) can be recommended, considering that its weight-reducing and anti-inflammatory effects as well as its symptom-reducing effects have been described” (94.7% consensus).^2^ The ketogenic diet has been demonstrated to substantially reduce body weight, BMI, pain, and other anthropometric measurements, while also improving the overall quality of life in patients with lipedema.^35^

**Table 1.** Studies on the Effects of the Ketogenic Diet on Lipedema

Study group	Diet protocol	Results	Reference
32-y-old woman diagnosed with lipedema	>1-y ketogenic diet with 200-250 kcal energy deficit (protein: 30 E%, dietary fat: 66 E%, carb: 4 E%)	Significant weight loss (−41 kg), net reduction in body circumferences, and perception of less pain	Cannataro et al. (2021)^86^
9 women with lipedema (BMI: 36.7 ± 4.5 kg/m^2^; age: 46.9 ± 7 y)	7-wk eucaloric ketogenic diet (fat: 70-75 E%, carb: 5-10 E%, protein: 20 E%) and then 6-wk Nordic diet	Significant body weight loss at end of 7th wk (Δ − 4.6 kg, *P* < .001) and 13th wk (Δ − 4.1 kg, *P* < .001) Significant decrease in pain at end of 7th wk (*P* = .018), but pain returned to baseline levels at wk 13	Sørlie et al. (2022)^54^
91 women with lipedema (age: 43.2 ± 12.8 y)	Two caloric-restricted diet groups for 16 wk (designed with Mediterranean style) Low-carb high-fat diet group (dietary fat: 72.3 E%, carb: 6.1 E%) (n = 46) and medium carb medium fat diet group (dietary fat: 39.1 E%, carb: 39.1 E%) (n = 45)	Significant decrease in anthropometric measurements in both diet groups. Nonetheless, low-carb, high-fat diet group experienced a larger reduction in body weight, body fat, and lower limb circumferences.	Jeziorek et al. (2022)^40^
48 women: lipedema (*n* = 24) and overweight/obese (*n* = 24)	All participants received diet low in carbs and high in fat, but with caloric restriction (carb intake in both groups <50 g/d, designed with Mediterranean style) for 7 mo	Significant decrease in body weight and serum triglyceride levels in both groups. Improvement in glucose tolerance and fasting insulin levels in both groups but less pronounced in lipedema group.	Jeziorek et al. (2023)^41^
52 women: lipedema (*n* = 28) and overweight/obese (*n* = 24)	+All participants received diet lo in carbs and high in fat, but with caloric restriction (<50 g/d carb and designed with Mediterranean style) for 7 mo	Significant decrease in body weight in both groups (∼12.9%) and pain in lipedema group.	Jeziorek et al. (2023)^39^
22 women with lipedema (mean age 46 y)	10-wk treatment groups: modified Mediterranean ketogenic diet (<30 g/d carbs, 20%-25% protein, 70% dietary fat) combined with carboxytherapy (*n* = 8), modified Mediterranean ketogenic diet alone (*n* = 8), carboxytherapy alone (*n* = 6)	Combination of ketogenic diet and carboxytherapy group recorded best results, with improvements in pain, skin texture, and body composition.	Di Renzo et al. (2023)^38^

Abbreviations: BMI, body mass index; carb, carbohydrate; E%, percentage total energy intake.

The Mediterranean version of the ketogenic diet, which has become widespread recently, preference for olive oil as a fat source, limitation of saturated fatty acids, preference for protein sources with high biological value, and inclusion of fruits and vegetables in every possible meal (seasonal, low glycemic index).^91^ The standard care guideline for lipedema published in the United States also recommends plant-based, low-carbohydrate diets for lipedema and that the nutritional plan minimize post-meal insulin and glucose fluctuations and be sustainable in the long term.^29^ In studies by Jeziorek and colleagues, a low-carbohydrate and high-fat diet (ketogenic diet) was designed in accordance with the Mediterranean diet. In their study conducted on 91 women with lipedema, participants were divided into 2 diet groups: a low-carbohydrate, high-fat diet and a moderate- carbohydrate, moderate-fat diet. At the end of 16 weeks, there was a significant decrease in anthropometric measurements in both diet groups. However, the decreases in body weight, fat mass, and lower limb circumferences was greater in the low-carbohydrate, high-fat diet group.^40^ In studies evaluating low-carbohydrate, high-fat diets in groups of women with lipedema and overweight/obesity, a significant decrease in body weight^39^^,^^41^ and pain^39^ was recorded. However, improvement in glucose tolerance and fasting insulin levels was less pronounced in the lipedema group.^41^ In a pilot study by Di Renzo et al. related to lipedema and the modified Mediterranean ketogenic diet, 22 patients with lipedema were divided into 3 groups with a 10-week treatment period: modified Mediterranean ketogenic diet and combined carboxytherapy group, modified Mediterranean ketogenic diet group, and carboxytherapy group. At the end of the study, the best results were obtained in the combined treatment group. Both the combined group and the diet group showed significant decreases in body weight, body fat mass, and pain.^38^

Although study results have shown the positive effects of the ketogenic diet in weight loss and pain reduction, we need methodologically strong studies with larger samples on the effectiveness of the ketogenic diet in the treatment of lipedema. In addition, the ketogenic diet is a nutritional model that can cause headaches, fatigue, and gastrointestinal discomfort in the short term^92^ and nutritional deficiencies,^93^ dyslipidemia, carnitine deficiency, kidney stones, and osteoporosis in the long term.^94^ To lessen the symptoms of short-term, maintaining electrolyte balance and staying well hydrated are essential, and make sure you’re getting enough nutrients throughout the early stages of the ketogenic diet.^95^ Also, it is important to provide nutrient supplementation to prevent deficiencies and increase mono- and polyunsaturated fatty acids in the diet to control dyslipidemia.^96^ Therefore, a modified Mediterranean-ketogenic diet intervention may be more beneficial in lipedema.

#### The Very Low-Calorie Ketogenic Diet

**Figure 3.**
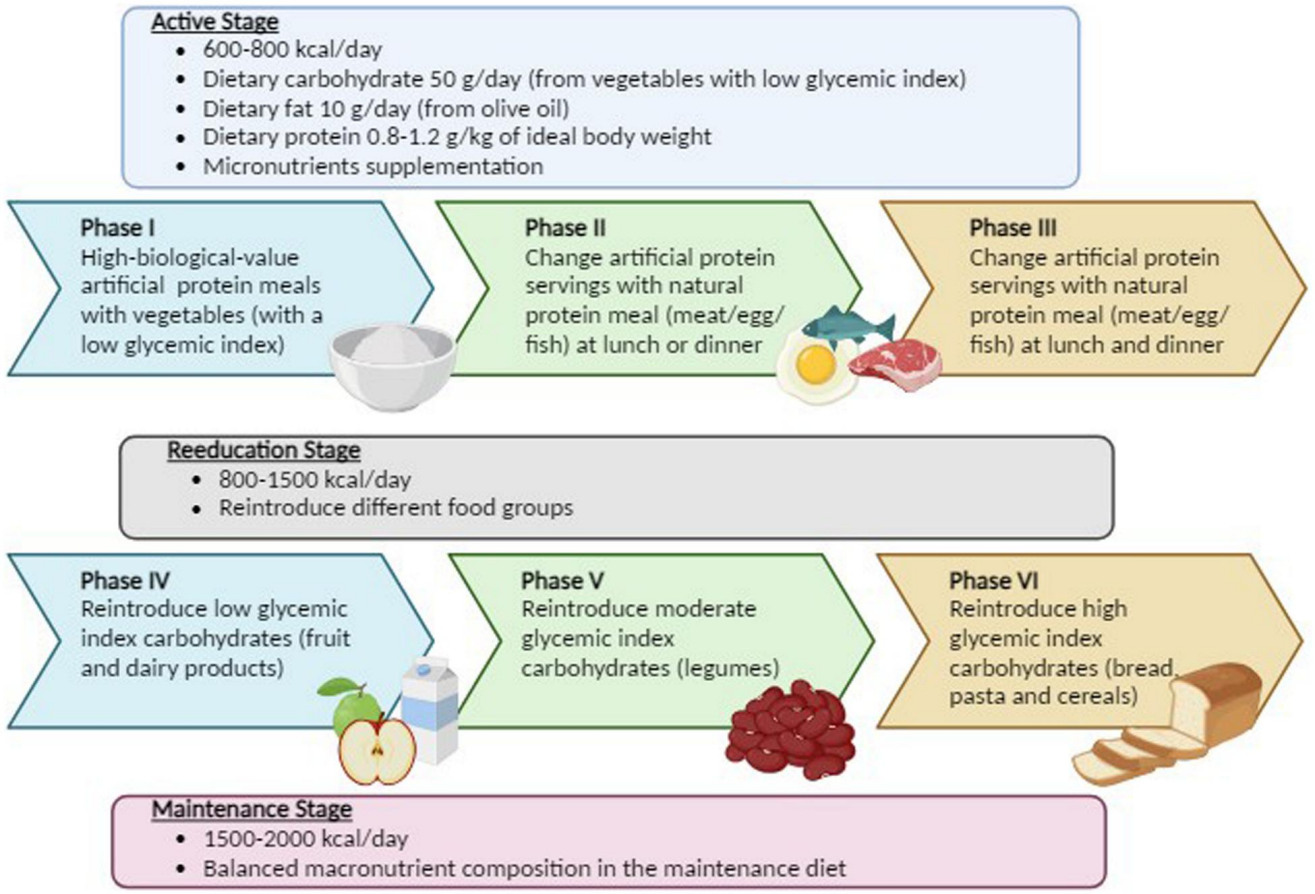
Phases of the Very Low-Calorie Ketogenic Diet (VLCKD) Protocol

The ketogenic diets used to treat obesity, the low-calorie ketogenic diet and very low-calorie ketogenic diet (VLCKD), include both calorie restriction and a decrease in carbohydrate intake.^36^ The low daily calorie intake of 700-800 kcal, carbohydrate restriction to 30-50 g/d (about 13% of total energy intake), 30-40 g/d (approximately 44%) increase in dietary fat, and approximately 1.2-1.5 g/d of proteins per kilogram of body weight (approximately 43%) are the hallmarks of the VLCKD strategy.^97^ There are phases to follow in the VCLKD protocol (summarized in Figure 3). The active phase is continued until the patient loses 80% of body weight. The duration may be 8-12 weeks, although it varies individually.^98^ The VLCKD has been reported to be more suitable for patients with lipedema because ketosis provided by the increase in beta hydroxybutyrate levels can inhibit NLRP3 activation, inflammation, and fibrosis tissue formation. In addition, body weight loss and fat tissue loss can be achieved, and protein catabolism is prevented.^36^

Severe obesity, preoperative time before BS, and obesity linked to insulin resistance are the primary indications for the use of the VLCKD. The VLCKD is known to lead to significant weight loss and improvement in body composition parameters and glycemic and lipid profiles in the short, medium, and long term.^98^ Findings such as typical resistance to weight loss,^1^^,^^29^ abnormal lipid metabolism and insulin resistance in lipedema^21^ predicted that VLCKD may be effective in this patient group. However, to our knowledge no published study to date has reported on the effect of VLCKD on lipedema. Two studies in the literature have been described as VLCKD studies. However, because one of the mentioned studies involved a eucaloric ketogenic diet intervention^54^ and the other involved a 200-250 kcal reduction in dietary energy,^86^ these studies cannot be defined as VLCKD studies. There is a need to plan studies on the effects of VLCKD in lipedema. However, due to possible side effects, follow-up is essential in this dietary intervention. One potential concern associated with VLCKD is the loss of lean tissue mass during rapid weight reduction.^99^ Maintaining adequate dietary protein intake—particularly rich in leucine and high-quality whey proteins—along with addressing vitamin D insufficiency or deficiency through supplementation play crucial roles in preserving lean body mass throughout the intervention.^100^ The Italian Society of Endocrinology recommends the use of VLCKD even in the context of sarcopenic obesity without relevant concerns regarding loss of lean body mass.^97^

#### Low-Carbohydrate Diet

Because carbohydrate intake is generally <50 g/d in the ketogenic diet,^84^ studies reporting higher carbohydrate intakes (75 g) were evaluated as low-carbohydrate diet models. Certain studies on the effects of a low-carbohydrate diet on lipedema are given in Table 2. In 3 studies by Lundanes and colleagues, different groups of patients with lipedema were randomized to receive either a 1200-kcal low-carbohydrate diet (total energy intake [%E]: carbohydrate: 25 %E, dietary fat: 55 %E) or a 1200-kcal control diet (carbohydrate 60 %E, dietary fat: 20 %E). After 8 weeks, in 1 of the mentioned studies, body weight and pain reductions were greatest on the low-carbohydrate diet,^43^ while in the other study, although both groups lost significant weight and fat mass, no significant difference was found between the groups.^42^ Additionally, a significant decrease in postprandial ghrelin level and an increase in postprandial fullness ratings and hunger in fasting were found in the low-carbohydrate diet group compared to the control.^44^ Hyperinsulinemia and insulin resistance are risk factors for adipose hypertrophy. Therefore, it is hypothesized that reducing carbohydrate intake in low-carbohydrate diets may decrease plasma insulin concentration and increase lipolysis in adipose tissue.^42^

**Table 2.** Studies on the Effects of a Low-Carbohydrate Diet on Lipedema

Study group	Diet protocol	Results	Reference
13 participants with lipedema age 18-75 y and BMI 30-45 kg/m^2^	Participants randomized to diet groups for 8 wk: Low carb diet (1200 kcal, carb: 25 E%, dietary fat: 55 E%) Low-fat, low-energy diet (control) (1200 kcal, carb: 60 E%, dietary fat: 20 E%)	Significant reduction in body weight and fat mass in both groups without differences between groups. Significant reduction in calf subcutaneous adipose tissue area and pain observed only in low-carb diet group.	Lundanes et al. (2024)^42^
Seventy participants with lipedema aged 18-75 y and BMI between 30 and 45 kg/m^2^	Participants were randomized to diet groups for 8 wk: Low carb diet (1200 kcal, carb: 25 E%, dietary fat: 55 E%) Low-fat, low-energy diet (control) (1200 kcal, carb: 60 E%, dietary fat: 20 E%)	Greater weight loss and pain reduction in low carb diet group.	Lundanes et al. (2024)^43^
55 participants with lipedema (age 47.9 ± 11.3 y, BMI 36.8 ± 5.1 kg/m^2^)	Participants randomized to diet groups for 8 wk: Low carb diet (1200 kcal, carb: 25 E%, dietary fat: 55 E%) Low-fat low-energy diet (control) (1200 kcal, carb: 60 E%, dietary fat: 20 E%)	Only low-carb diet group experienced decrease in postprandial ghrelin; this differed significantly from that of low-fat group. Low-carb diet group also reported increase in postprandial fullness ratings and hunger in fasting.	Lundanes et al (2025)^44^

Abbreviation: carb, carbohydrate; E%, percentage total energy intake.

Studies have shown that low-carbohydrate diets have positive effects on lipedema treatment;^42–44^ however, the current body of evidence is too limited to draw definitive conclusions. Studies should be planned to determine whether the ketogenic diet or low-carbohydrate diet has better results in lipedema treatment and which diet model may be more suitable for patients.

#### Other Dietary Patterns

Inflammatory processes, progressive interstitial fibrosis, and a higher percentage of M2-like macrophages are all linked to lipedema subcutaneous adipose tissue. Later stages of the disease are characterized by adipocyte hypertrophy and a more pro-inflammatory phenotype.^18^ Therefore, diet treatment that will provide weight loss and prevent inflammation may lead to well-being in patients with lipedema. The Mediterranean diet is an important dietary model for preventing inflammation due to the omega-3 fatty acids, vitamins, trace minerals, lycopene, and polyphenols it contains.^101^ The Mediterranean diet pattern may also help improve metabolic health by lowering metabolic endotoxemia and modifying the gastrointestinal flora.^102^ In the S2k guideline led by the German Society of Phlebology and Lymphology (DGPL), it was emphasized that “a (hypocaloric, if needed) Mediterranean diet may be recommended based on its anti-inflammatory properties” (100% consensus).^2^ However, there is 1 study in the literature evaluating the effects of the Mediterranean diet on lipedema. In the study by Di Renzo et al. 14 patients with lipedema and 15 controls without lipedema were followed with a hypocaloric modified Mediterranean diet with a 20% caloric restriction (carbohydrate: 40-45 E%, protein: 25-30 E%, dietary fat: 25-30 E%) for 4 weeks. At the end of the study, body weight and fat mass in the arms and legs significantly decreased in both groups, but the decrease in circumference measurements was significant only in the control group.^45^ In recent years, the MIND (Mediterranean– intervention [Dietary Approaches to Stop Hypertension] DASH neurodegenerative delay intervention) diet has been recommended as a nutritional model based on the Mediterranean diet. The most important feature of the MIND diet is the inclusion of foods with high antioxidant content, such as berries and green leafy vegetables.^103^^,^^104^ Therefore, the hypocaloric MIND diet may be used in addition to the Mediterranean diet in the treatment of inflammation in lipedema. Considering the risk of developing depression in patients with lipedema,^105^^,^^106^ the MIND diet appears to be a treatment option. A reduction in depressive and anxiety symptoms was reported with adherence to the MIND diet.^107^^,^^108^ However, we need to plan studies to evaluate the effectiveness of the MIND diet in the treatment of lipedema.

Inflammation caused by bacterial lipopolysaccharides resulting from increased intestinal permeability is also discussed in the pathogenesis of lipedema.^20^ This mechanism recalls small bowel bacterial overgrowth (SIBO), the term for increased colonization of the small intestine by bacteria, some of which are more common in the colon microbiota, which generally develops as a result of a decrease/impairment in the gastrointestinal antimicrobial defense mechanism, decreased gastrointestinal motility, and abnormalities in gastrointestinal anatomy.^109^ In addition, SIBO is very common during BS bypass procedures (43%).^110^ Intestinal barrier integrity degradation, increased intestinal permeability, elevated endotoxin levels, inflammatory response activation, and bacterial translocation from the colon to the small intestine are all possible outcomes of SIBO.^111^ Therefore, SIBO may be both a risk factor for the development of lipedema and a consequence of lipedema. Studies have shown that Western style diets, high in fat and simple carbohydrates, alter the intestinal microbiota composition and negatively impact intestinal permeability. Probiotics primarily contain *Lactobacillus* and *Bifidobacteria* to avoid intestinal endotoxemia. Additionally, vitamins—particularly D and A—are crucial for maintaining the integrity of the intestinal epithelium.^112^ Diets for SIBO treatment appear promising, but their practical relevance is limited by the scarce and poor quality of available trials. To achieve a balanced microbiome, a healthy diet full of whole foods and vegetables appears to be the best course of action.^113^

Another point mentioned about the issue of intestinal permeability in lipedema is gluten. In a study by Amato et al., the frequency of HLA-DQ8 (22.11%), either HLA-DQ8 or HLA-DQ2 (61.05%), both HLA-DQ8 and HLA-DQ2 (7.37%) in patients with lipedema was found to be higher than in the general population. A direct association cannot be established between lipedema and higher HLA allele frequency. On the other hand, the study established an indirect association between the increase in M2 macrophage levels in lipedema and the increase in intestinal permeability caused by gluten due to macrophage activation.^114^ Nonetheless, HLA gene positivity alone does not confirm a diagnosis of celiac disease or non-celiac gluten sensitivity. Therefore, in the absence of comorbid gluten-related immune-mediated conditions- such as celiac disease, dermatitis herpetiformis, gluten ataxia, wheat allergy, or non-celiac gluten sensitivity—a gluten-free diet should not be recommended for patients with lipedema. Adopting such a diet without a clear indication may present challenges, including social and psychological barriers, high cost, and the risk of nutritional deficiencies.^115^ The Standard of Care for Lipedema in the United States guideline highlights that, in a study of 100 individuals with lipedema, 74% had a history of eating disorders, with 12% experiencing periodic binge-eating episodes, 8% having bulimia, and 16% suffering from anorexia nervosa. This underscores the importance of addressing eating disorders as part of the comprehensive treatment for lipedema.^116^

### Inflammatory Processes Associated With Food Intolerances

Although the exact origin of lipedema is as yet unknown, inflammation is recognized to be a contributing factor.^29^ Dietary intake is one of the many factors that might induce lipedema, which results in recurring inflammatory symptoms.^114^ Certain foods can worsen lipedema beyond weight gain and cause inflammation, so diet is thought to play an important role in lipedema treatment.^45^

A Mediterranean diet approach and a ketogenic diet are often recommended, which recommend a lower carbohydrate intake in lipedema and have been proven effective.^86^ Reducing or eliminating gluten consumption and using an anti-inflammatory dietary strategy are noteworthy aspects of both dietary methods.^114^ Some studies suggest a possible association between gluten consumption and an increased abundance of non-celiac autoimmune and inflammatory diseases.^117^ By increasing intestinal permeability, gluten ingestion can help undigested food proteins and bacterial lipopolysaccharides move from the intestine into the bloodstream through tight junctions. This can cause the body to react to these compounds in an inflammatory and immunological manner. It is becoming more and more crucial to investigate the possible connection between gluten and the inflammatory disorder lipedema, especially in light of the concurrent rise in gluten consumption and the identification of inflammatory diseases like lipedema.^118^ Numerous dietary studies, including the modified Mediterranean diet known as the rare adipose disorder (RAD) diet,^119^^,^^120^ recommend gluten exclusion despite no confirmed link to lipedema.^121^

According to a recent study, there may be a connection between lipedema inflammation and human leukocyte antigen (HLA)-DQ2 or HLA-DQ8 (particularly when gluten is consumed). The findings of the study imply that there may be a cause-and-effect relationship between lipedema and HLA-associated inflammation, even if this has not yet been established. Additionally, all patient groups who received dietary counseling saw a significant improvement after avoiding gluten-rich carbohydrates.^114^ This effect seems to occur because consuming gluten may make the body more permeable and possibly boost the activation of macrophages. The increased prevalence of M2 macrophages in lipedema could start the angiogenesis and fibrosis processes.^118^^,^^122^^,^^123^ It has been demonstrated that adipose tissue macrophages, which control mitochondrial activity and energy metabolism, are crucial in lipedema.^118^ However, there is insufficient evidence to routinely recommend a gluten-free diet in lipedema. Therefore, dietary interventions should always be considered with a careful and holistic approach and should be taken into consideration in individuals reporting symptoms, especially in relation to dietary gluten.

Aspirating lipidemic fat during liposuction does not treat the underlying intolerance, and the surgery may cause visceral fat to grow. This process might increase inflammation in the brain and refocus the attention of gluten-induced inflammation. Brain shrinkage is more strongly correlated with visceral fat than with age, BMI, hypertension, and type 2 diabetes.^114^^,^^124^ The fundamental issue is not addressed by this intervention, despite the fact that it might reduce limb pain.^125^^,^^126^

It is important to evaluate inflammatory stimuli on an individual basis.^121^ For example, in the case of gluten sensitivity, gluten should be eliminated.^120^ In the same way, if there is lactose intolerance, it should be known that this may affect the intestinal microbiota. It should be emphasized that it may be a pro-inflammatory stimulus.^127^

Current literature suggests that the diagnosis of lipedema does not, by itself, require the elimination of any food or food group from the diet. Most adverse reactions to foods or nutrients are self-reported and are not based on validated diagnostic tests.^128^ Since no reliable laboratory tests are available for diagnosing food intolerances, the most accurate approach relies on a detailed clinical history and careful symptom assessment. Following an individualized evaluation, elimination diets should be implemented only in cases of symptomatic intolerance to a specific food or food group.

### Nutritional Biotics

Chronic low-grade inflammation resulting from intestinal lipopolysaccharide (LPS) translocation has important effects on fat cell function (for example, hindering insulin signaling and lipid metabolism, encouraging insulin resistance, and causing metabolic disorders). Therapeutic strategies targeted at reducing inflammation and its metabolic effects in lipedema and other comorbidities linked to it (such as obesity, hypothyroidism, allergies and asthma, polycystic ovary syndrome, and lymphedema, which are also linked to inflammation, LPS, and endotoxemia) would benefit from an understanding of this intricate system.^20^ One possible strategy to reduce chronic inflammation in lipedema may be to use therapeutic measures to restore intestinal barrier integrity by decreasing LPS translocation.^129^ Research is being done on methods including using anti-inflammatory drugs, dietary changes, probiotics, and prebiotics to alter the gut microbiota.^130^ The translocation and systemic inflammation of LPS may be decreased by therapeutic approaches that restore a healthy microbiota, such as probiotics, prebiotics, exercise, and a balanced diet.^131^

The potential of nutritional biotics to reduce inflammation and modulate the immune response offers a promising approach to the management of lipedema. Given the role of chronic inflammation in the pathophysiology of lipedema, we believe that nutritional biotics (with potential safety issues) can be considered as a potential complementary treatment in the management of this disease. However, no specific clinical studies have been found that directly examine the relationship between lipedema and nutritional biotics. Therefore, comprehensive and specific research needs to be conducted on this subject.

### Dietary Supplements in the Management of Lipedema

Since there is no definitive nutritional or effective pharmacological treatment for lipedema, focusing on nutrition and indicated dietary supplements seem important in the long-term management of lipedema. Dietary strategies that can treat and/or mitigate the symptoms and problems of lipedema may result in vitamin and mineral losses. The usefulness of any dietary supplement in treating lipedema has not been established, based on evidence from the current literature.^46^ Supplements and anti-inflammatory diets seem to play a significant role in the chronic inflammation seen in lipedema patients. Targeting inflammation may enhance patient quality of life because it can exacerbate pain and discomfort.^18^ This section examines micronutrients and molecules that may benefit lipedema symptoms and highlights deficiencies caused by medical nutrition treatments.

#### Vitamin C

Vitamin C may contribute to the inflammation and connective tissue management of lipedema through its antioxidant effects and support for collagen synthesis.^46^^,^^132^^,^^133^ One case report has demonstrated positive results after using 1000 mg of vitamin C.^86^

#### Vitamin B12

Vitamin B12 is thought to help manage the painful neuropathic component that becomes increasingly evident in lipedema.^46^ One study has emphasized that vitamin B12 is also effective in treating pain, especially neuropathic pain.^134^ Considering plasma values, 500-100 mcg of B12 supplementation is recommended.^46^

#### Magnesium

The mineral magnesium, which is widely used by individuals with lipedema, is not considered effective because it does not show a direct effect on the complications of lipedema according to the limited evidence available.^46^

#### Selenium

Results of a retrospective cross-sectional research study demonstrated that people with lipedema had a selenium shortage.^135^ However, this micronutrient shortage may not be directly linked to lipedema because it is equally prevalent in the healthy population (connected to the location). It is advised that plasma selenium levels be checked and supplements be taken as needed because selenium is crucial for the healthy operation of the immune system and the control of free radicals.^46^

#### Omega-3 fatty acids

The anti-inflammatory effect of omega-3 fatty acids ^136^ may be important due to the inflammatory process in lipedema. Therefore, the effects of the omega-3 fatty acids docosahexaenoic acid (DHA) and eicosapentaenoic acid (EPA) are important.^46^ One study has highlighted that DHA and EPA supplements may support adipocyte health through a mechanism associated with reduced activation of macrophages and, therefore, lower pro-inflammatory cytokine secretion.^137^ Except for the case report by Cannataro et al.^86^ on omega-3 fatty acid supplementation, we have not found any studies directly studying lipedema.

In addition, DHA and EPA supplementation are also important for their synthesis of inflammation-modulating mediators such as resolvin and maresin and the protection family in general. Although the mechanism of action is not fully known, it has been stated that this process may be related to the transient receptor potential (TRP) channel, which plays a role in transmitting pain-related signals.^138^^,^^139^ This finding may be a significant feature, as inflammation can be an important trigger of pain in lipedema, and at least 80% of individuals affected by lipedema present a painful component. A daily intake of at least 1 g of DHA and EPA may be recommended to alleviate inflammation and the painful component.^46^

#### Polyphenols

Polyphenols may be effective in managing the inflammatory process observed in lipedema due to their antioxidant properties.^140^ As polyphenols regulate nuclear factor kappa B (NF-κB) activity, they are recommended for consumption because of their strong influence on the synthesis of inflammatory mediators and free radicals.^141^^,^^142^ Overall, the antioxidant and anti-inflammatory properties of polyphenols make them promising candidates for supporting the management of lipedema-associated inflammation.

Curcumin is a polyphenol that is effective both as an antioxidant and an NFkB regulator.^140^ The nuclear factor erythroid 2-related factor 2 (Nrf2), a mediator that helps control and manage reactive oxygen species (ROS), is another mechanism that curcumin activates. Due to these properties, Cannataro et al. reported that curcumin has positive effects in the management of the inflammatory process seen in lipedema. These researchers have recommended a polyphenol-rich diet, such as the Mediterranean diet, for patients with lipedema (daily intake of 100-150 mg of polyphenols from multiple sources).^46^

#### Diet-related micronutrient deficiencies and supplements

While low-carbohydrate nutritional approaches such as the ketogenic diet pose a risk of causing serious micronutrient deficiencies in lipedema patients, antioxidant and anti-inflammatory diets (eg, the Mediterranean diet), which have an important place in medical nutrition therapy in lipedema, can adequately meet the body’s vitamin and mineral requirements thanks to their high vegetable and fruit content.^143^ It has been shown that after 3 months of the ketogenic diet, the body’s manganese, vitamin D, and E requirements cannot be met.^144^ Monitoring vitamin D and calcium levels at the beginning and end of the ketogenic diet is recommended.^36^ Any supplements that improve antioxidant status, such as vitamin C, which can be very scarce in the ketogenic diet, should be considered supplements.^121^ It is also worth considering N-acetyl-cysteine (NAC) and its polyphenol and anthocyanin pool, which is very useful in improving the response to free radicals,^140^^,^^145^^,^^146^ as well as diosmin, which is often used to support lipedema.^147^ These supplements should also be considered in a ketogenic dietary pattern for the management of inflammation that recommends limited or no fruit intake and only allows some vegetables.^121^

It has been shown that a low-carbohydrate diet may increase urinary calcium excretion, pose a risk for bone loss,^148^ and may be inadequate in meeting the body’s micronutrient needs.^149^ A systematic review found that a low-carbohydrate diet decreased intake of thiamine, folate, magnesium, iron, and iodine.^150^

The inflammatory condition brought on by lipedema will increase if micronutrient shortages result from dietary strategies that might be employed as treatment. As a result, the application of dietary supplements with anti-inflammatory qualities has to be assessed according to the patient requirements. For instance, ascorbic acid, an antioxidant and anti-inflammatory agent, has been shown to alleviate pain in patients with inflammation induced by lipedema adipose tissue.^151^ Additionally, these patients should have their levels of micronutrients with anti-inflammatory qualities monitored, such as zinc, which lowers oxidative stress and suppresses inflammation; selenium, which helps to alleviate the inflammatory process by participating in the structure of some antioxidant enzymes; and vitamin D, which modulates systemic inflammation.^152–154^

Furthermore, according to international guidelines, patients should be supported with micronutrients (vitamins like B-complex vitamins, vitamins C and E, and minerals like potassium, sodium, magnesium, calcium, and omega-3 fatty acids) when following a very low-calorie diet plan.^36^^,^^155^ Although some supplements stand out as potential options for treating lipedema, more scientific studies are needed for their effectiveness and safety. Some nutritional supplements, their dosages, and recommended levels are given in Table 3.

**Table 3.** Dietary Supplements and Dosages in Lipedema Based on Current Literature^46^^,^^86^

Supplement	Biochemical action	Dosage	Suggestion
DHA and EPA	Anti-inflammatory	1-2 g/d	Suggested
	Pain relieving		
Vitamin C	Antioxidant	500-1000 mg/d	Suggested
	Support of collagen synthesis		
Vitamin B_12_	Pain relieving	500-1000 mcg	Evaluate its use
	Neuropathy treatment		
Magnesium	General health	300-400 mg/d	Evaluate its use
	Muscle trophism		
	Pain management		
Selenium	Immune system	45-60 mcg/d	Evaluate its use
	ROS scavenging		
Polyphenols	Anti-inflammatory	100-200 mg/d	Evaluate its use
	Antioxidant		
Vitamin D	Adipose tissue health	2000 IU/d	Evaluate its use
	Immunomodulation		

Abbreviations: DHA, docosahexaenoic acid; EPA, eicosapentaenoic acid; ROS, reactive oxygen species.

It should be noted that supplements addressing these micronutrient deficiencies should only be used when appropriately indicated and under the supervision of a dietitian and a physician. The benefit–risk balance of each supplement should be carefully evaluated, and a cautious approach should be taken to correct deficiencies.

#### Fat-burning supplements and foods

Green tea, caffeine, chromium, carnitine, conjugated linoleic acid (CLA), ephedrine, pyruvate, yohimbine, and chitosan are a few examples of nutrients that burn fat. Through several biochemical pathways, fat-burning substances can promote weight loss by boosting metabolism and decreasing appetite.^47^ Similarly, certain foods can support the fat-burning process through these mechanisms.^156^ Both mechanisms are presented in Table 4. In lipedema, supplements and foods that can help reduce body fat by reducing fat absorption and regulating fatty acid metabolism may play an important role. In particular, components that increase fat burning with thermogenic effects and suppress hunger may be useful in treating lipedema.

**Table 4.** Some Mechanisms of Fat-Burning Supplements and Foods

Supplements/foods	Mechanism
Supplements	
Chitosan (found in crustaceans)	Reduces fat absorption
l-Carnitine (chemical catalyst synthesized by human kidneys, brain and liver)	Participates in transport of fatty acids into mitochondria during breakdown of fats
Chromium (trace mineral found in meat, grains, and nuts)	Reduces insulin resistance
Ephedrine (***Ephedra sinica*** plant)	Stimulates sympathetic neuronal activity
Synephrine (citrus fruits)	Stimulates thermogenesis
Pyruvate (intermediate of glycolysis)	Reduces appetite and fatigue, increase energy levels and muscle glycogen stores
Conjugated linoleic acid (meat and dairy products)	Transports dietary fats into cells for lipolysis
**Foods**	
Good oils (avocado, hazelnut, fish, vegetable oils)^156^	Supports fat burning through β-sitosterol, oleic acid, and omega-3 content
Medium-chain triglycerides (butter, palm oil, and coconut oil)^157^	May suppress appetite and stimulate body fat loss because they are easily digested, absorbed, and used directly for energy
Meat and dairy proteins^156^	1st mechanism: Stimulate glucagon release, keeping insulin production low and allowing body to access fat more effectively and use it as a fuel source
	2nd mechanism: Accelerate lipolysis by promoting growth-hormone secretion

#### Edema-reducing agents

Two agents can be mentioned in this category: bromelain and serratiopeptidase.

Bromelain is a characteristic proteolytic enzyme of pineapple. Cannatora et al. have suggested that bromelain should be tested as a potential option to manage edema in lipedema due to its fibrinolytic and lipid-dissolving effects on clots.^46^ However, no specific study has been found that has investigated bromelain for this purpose. Additionally, bromelain may be beneficial for lipedema due to its immunomodulatory and antimicrobial properties.^158^ It is a widely used and relatively safe supplement but its effectiveness remains to be tested as its mechanism of action is not yet fully understood.^46^

Serratiopeptidase, a proteolytic enzyme produced by the bacterium *Serratia marcescens*, is sensitive to gastric acidity. Due to its proteolytic and fibrinolytic effects, it is also considered a potential agent for reducing the edema commonly observed in lipedema.^159^

#### Possible supplements related to amino acid metabolism

In a study of lipedema patients, some metabolic changes related to amino acid levels were reported. These changes indicated possible disturbances in amino acid metabolism in patients with lipedema.^160^ Accordingly, histidine and phenylalanine levels were low in the lipedema group, a condition that may affect protein synthesis, energy production, and neurotransmitter regulation.^161^ In the study by Kempa et al., pyruvate levels increased in lipedema patients independently of BMI. These researchers reported that high lipedema pyruvate levels may indicate a disruption in the citric acid cycle.^160^ Pyruvate metabolism meets the energy and storage demands of fat cells in response to nutritional and physiological stimuli. An alteration in pyruvate metabolism in fat cells can lead to the accumulation of triglycerides.^162^ In the same study, decreased levels of acetic acid, glycine, glutamine, and lactic acid in the lipedema group suggested disruption in multiple metabolic pathways.^160^ Decreased acetic acid may indicate altered lipid metabolism, particularly fatty acid oxidation or synthesis.^163^ Serum levels of glycine are positively correlated with subcutaneous fat tissue and negatively correlated with visceral fat tissue,^164^ suggesting a potential role for this amino acid in the distribution of fat tissue.^160^ Finally, glutamine has been reported to play a role in nitrogen metabolism, energy production, and neurotransmitter synthesis, and it has been predicted that decreased levels of glutamine may reflect changes in these processes.^160^ Glutamine can reduce fat tissue mass and inflammation.^165^ These findings raise the question of whether these amino acid supplements could be used as a therapeutic approach for patients with lipedema. But at this point, it is impossible to provide a definitive answer because to our knowledge there are no clinical studies in literature on the regulation of these amino acid levels in lipedema patients through nutrition and/or supplementation.

## FUTURE DIRECTION

Although the precise etiology of lipedema remains unknown, growing evidence highlights the potential importance of nutrition in managing symptoms and improving patient quality of life. However, current randomized controlled trials on the relationship between lipedema and nutrition remain limited. Future studies and clinical approaches should focus on personalized nutrition strategies. Advances in precision medicine and nutrigenomics may lead to tailored dietary recommendations based on a patient metabolic profiles, gut microbiota, and genetic composition. Identifying dietary components that contribute to inflammation and fat accumulation is essential for developing individualized nutritional treatments. Because inflammation is believed to play a role in lipedema progression, future studies should explore the efficacy of anti-inflammatory diets, particularly modified Mediterranean–ketogenic diets. Weight management is a key consideration for lipedema patients. To ensure effective weight and fat loss, dietary plans must accurately determine energy requirements. Therefore, formulas for calculating BMR should be developed and validated. Research should also focus on the impact of different macronutrient ratios (carbohydrates, fats, and proteins) on lipedema symptoms and fat distribution. Additionally, examining the role of essential micronutrients could help refine dietary guidelines. Clinical trials are necessary to determine which dietary patterns offer the greatest benefits for lipedema patients. However, many existing studies have been conducted over short durations with small sample sizes. While small-scale or short-term studies may yield quick results, their reliability and predictive power may be limited. Therefore, these findings should be integrated into methodologically robust, longitudinal studies with larger patient groups. Studies on the gut microbiota in lipedema are particularly valuable, as recent research suggests a strong link between metabolic health and the gut microbiome. Investigating how the gut microbiome influences inflammation and fat distribution in lipedema could pave the way for probiotic and prebiotic treatments that enhance disease management. Recently, microRNAs (miRNAs) identified as being released from adipose tissue into the bloodstream have been proposed as potential future biomarkers for understanding the biochemical mechanisms underlying the pathogenesis of lipedema. Also, investigating the effects of targeted dietary interventions on miRNA profiles may contribute to the development of more personalized approaches in lipedema management. Finally, research on the relationship between nutrition and lymphatic health is crucial, as lipedema and lymphatic dysfunction are closely interconnected.

## CONCLUSION

Lipedema management is a multifactorial process that requires a comprehensive, multidisciplinary approach. In the treatment of lipedema, the main goal should be to eliminate/improve the signs and symptoms and prevent complications. Bariatric surgery (BS) is not an effective solution for the treatment of lipedema. Accurate calculation of BMR in lipedema patients is of great importance for implementing appropriate calorie-restricted diets. Therefore, a new formulation has been developed to calculate BMR more accurately in lipedema patients when the gold standard indirect calorimetry (IC) is unavailable. BMI and body weight are considered imprecise measures of obesity in lipedema patients due to the disproportion in the lower extremities.

When looking at current studies in the literature, although no diet approach has been proven to be applicable in lipedema patients, those that are stated to be more suitable than other diets are in particular the ketogenic diet and the Mediterranean diet, as well as anti-inflammatory diets due to their low carbohydrate content and anti-inflammatory properties. It has been seen that the alleviating effects of lipedema are generally realized through low carbohydrate consumption and antioxidant and anti-inflammatory processes. Therefore, in the medical nutrition treatment of lipedema, although it is not a proven approach, the aim should be to create nutritional patterns that support ketosis formation and have antioxidant and anti-inflammatory content. It is thought that the modified Mediterranean–ketogenic diet intervention may be more beneficial. It should be noted that lipedema may be associated with inflammatory processes and that food intolerances may play a role in some patients. After a personalized assessment, eliminating gluten and lactose from the diet may be recommended for intolerance. However, no elimination diet has been proven to be effective on lipedema to date. In addition, nutritional recommendations to prevent SIBO after BS should be considered.

The effectiveness of nutritional supplements in managing lipedema has not been proven. Vitamin and mineral levels, which may be related to the medical nutrition treatments applied and have an important place in the prognosis and clinical picture of the disease, should be closely monitored. Although the effect of micronutrient supplements has not been proven in individuals with lipedema, if micronutrient deficiency is present or occurs during the treatment process, appropriate supplement use should be considered. Preventing micronutrient deficiencies should be important in allowing dietary approaches appropriate for lipedema patients to be applied for a longer period without vitamin and mineral deficiencies. While some ingredients can be used based on evidence, it is too early to say for sure about many supplements that do not yet have sufficient scientific support and remain at a speculative level. Whenever possible, it is recommended that essential nutrients be obtained through an adequate and balanced diet rather than relying solely on supplements. While the observed positive effects reported in the existing literature are promising, further scientific validation is needed to confirm their effectiveness.

Future studies will contribute significantly to determining the effectiveness of individualized nutritional approaches for lipedema management and developing optimal nutritional strategies in these patients.
